# Doctor, what is my risk of bleeding after cardiac surgery while on combined anticoagulant with antiplatelet therapy? A validated nomogram for risk assessment

**DOI:** 10.3389/fphar.2024.1528390

**Published:** 2025-01-07

**Authors:** Haolong Han, Hang Xu, Jifan Zhang, Weihui Zhang, Yi Yang, Xia Wang, Li Wang, Dongjin Wang, Weihong Ge

**Affiliations:** ^1^ School of Pharmacy, Faculty of Medicine, Macau University of Science and Technology, Macau, China; ^2^ Department of Pharmacy, Nanjing Drum Tower Hospital Clinical College of Nanjing University of Chinese Medicine, Nanjing, China; ^3^ Nanjing Foreign Language School, Nanjing, China; ^4^ Department of Pharmacy, Nanjing Drum Tower Hospital, School of Basic Medicine and Clinical Pharmacy, China Pharmaceutical University, Nanjing, China; ^5^ School of Business, Nanjing University, Nanjing, China; ^6^ Department of Cardiothoracic Surgery, Nanjing Drum Tower Hospital Clinical College of Nanjing University of Chinese Medicine, Nanjing, China

**Keywords:** coronary artery bypass grafting, surgical valve surgery, anticoagulation combined with antiplatelet, nomogram, bleeding risk

## Abstract

**Background:**

Patients with comorbid coronary artery disease and valvular heart disease usually undergo coronary artery bypass grafting alongside valve replacement or ring repair surgeries. Following these procedures, they typically receive a combination of anticoagulation and antiplatelet therapy, which notably heightens their bleeding risk. However, Current scoring systems provide limited predictive capability.

**Methods:**

A total of 500 adult patients treated with anticoagulation plus antiplatelet therapy after cardiac surgery were randomly divided into the training set and the validation set at a ratio of 7:3. Predictive factors were identified using univariate logistic regression, LASSO regression and multivariable analysis. Various models were developed, validated and evaluated by using methods including ROC curves, calibration curves, the Hosmer-Lemeshow test, net reclassification improvement (NRI), integrated discrimination improvement (IDI) index, decision curve analysis (DCA) and clinical impact curves (CIC).

**Results:**

Mod2 showed the best performance (AUC of validation set = 0.863) which consists of 8 independent predictive factors (gender, age > 65 years, diabetes, anemia, atrial fibrillation, cardiopulmonary bypass time, intraoperative bleeding and postoperative drainage), with a significantly higher AUC compared to Mod1 (only preoperative factors) and Mod3 (the HAS-BLED scoring model). NRI and IDI analyses further confirmed the superior predictive ability of Mod2 (NRI < 0.05, IDI < 0.05). Both DCA and CIC indicated that Mod2 exhibited good clinical applicability.

**Conclusion:**

This research established and validated a nomogram model incorporating eight predictive factors to evaluate the bleeding risk in patients who receive anticoagulation combined with antiplatelet therapy following cardiac surgery. The model holds significant potential for clinical applications in bleeding risk assessment, decision-making and personalized treatment strategies.

## 1 Introduction

Coronary artery disease (CAD) is a common cardiovascular disorder that poses serious life-threatening risks and is usually treated with coronary artery bypass grafting (CABG) (Ye et al., 2023). Similarly, valvular heart disease (VHD) significantly contributes to physical impairment, diminished quality of life and reduced lifespan, which is treated by valve replacement or ring repair surgeries (Sun et al., 2023). In patients with both CAD and VHD, the indications for combined CABG and surgical valve operations are increasing, which accounts for about 7% of all major cardiac surgeries (Bowdish et al., 2020).

Current clinical guidelines recommend that patients who undergo CABG combined with valve surgery receive a combination of anticoagulant and antiplatelet therapy—typically involving a vitamin K antagonist (VKA) alongside aspirin or clopidogrel (Valgimigli et al., 2018; Stein et al., 2004; Rugivarodom and Charatcharoenwitthaya, 2020). Studies indicate that the bleeding risk for patients treated solely with vitamin K antagonists (VKA) typically falls between 10% and 20%. Likewise, patients on antiplatelet therapy with either aspirin or clopidogrel experience a similar bleeding risk (Lopes et al., 2019). However, when anticoagulation is combined with a single antiplatelet agent, the bleeding risk increases by 20%–60% compared to those receiving either anticoagulation or antiplatelet therapy alone (Dewilde et al., 2013; Fortuni et al., 2018; Hansen et al., 2010; Vranckx et al., 2019). Major bleeding can result in serious physiological and clinical repercussions, with studies identifying it as a significant predictor of mortality in patients (Doyle et al., 2009). We should also take mild bleeding into account seriously, as even minor bleeding can lead to the cessation of antithrombotic therapy, thereby heightening the risk of thrombotic complications (Roy et al., 2008). Accordingly, developing a predictive model for bleeding risk is vital for helping clinicians create tailored bleeding prevention strategies for these patients.

Nomograms are widely utilized as prognostic assessment tools in the medical field (Zuo et al., 2023). They combine various prognostic and determining variables to produce individual numerical probabilities of clinical events, which satisfies the need for integrated biological and clinical models and advancing personalized medicine. Nomograms with the user-friendly digital interface allows for rapid calculations while improving accuracy and making prognostic information more accessible than traditional staging methods. Consequently, the prognoses generated by nomograms can be easily integrated into clinical decision-making (Balachandran et al., 2015).

Against this backdrop, this study aims to develop a practical nomogram for predicting the bleeding risk in patients undergoing anticoagulation combined with antiplatelet therapy after cardiac surgery. We will compare our risk prediction model with the HAS-BLED score which is widely used in clinical practice. While the HAS-BLED score is somewhat effective in differentiating between high-risk and low-risk bleeding patients, research indicates that its predictive ability is often limited (Shoeb and Fang, 2013; Abu Assi et al., 2011; Gulec, 2014). Therefore, evaluating the differences between the HAS-BLED score and our model will provide crucial insights for clinicians to enhance their decision-making process.

## 2 Methods

### 2.1 Statement

The data from this study can be accessed directly from the corresponding author upon reasonable request. This research complies with the ethical principles set forth in the *Declaration of Helsinki* and has been approved by the Ethics Committee of Nanjing University Medical School Affiliated Drum Tower Hospital (Approval No. 2024-546-01). Since this is a retrospective observational study, the ethics committee waived the need for written informed consent. Additionally, the reporting of this study follows the guidelines established by the Transparent Reporting of a Multivariable Prediction Model for Individual Prognosis or Diagnosis (TRIPOD) statement (Moons et al., 2015).

### 2.2 Study population

This study included patients aged 18 years or older who underwent cardiac surgery at Nanjing Drum Tower Hospital between January 2020 and June 2024 and received anticoagulant plus antiplatelet therapy postoperatively. These patients were treated with a combination of warfarin and aspirin for at least 3 months and received comprehensive education on anticoagulant management (Pernod et al., 2008).

**FIGURE 1:**
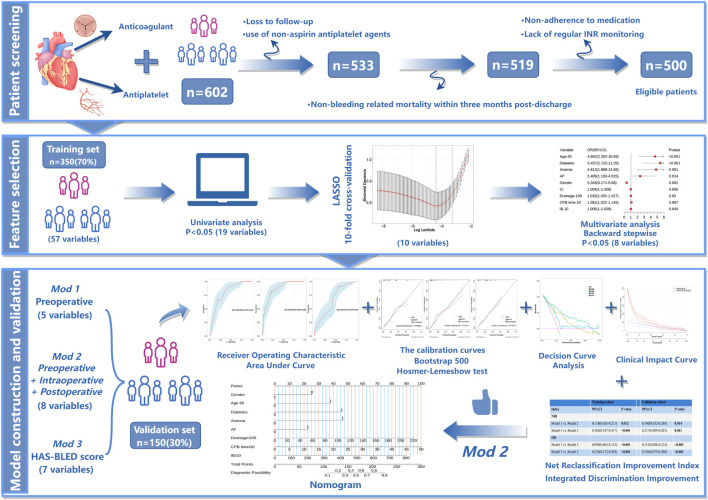
Patient screening flowchart and research methodology roadmap. (By Figdraw).

The exclusion criteria were: (1) Patients who did not follow the prescribed medication regimen within post-discharge 3 months and failed to regularly monitor their INR for warfarin dosage adjustments. (2) Patients who lost contact within 3 months after discharge. (3) Patients using non-aspirin antiplatelet medications (such as clopidogrel or ticagrelor). (4) Patients who died from non-bleeding causes within 3 months post-discharge (Figure 1).

### 2.3 Data collection

Patient data were collected by a specially trained researcher using the hospital’s electronic medical record system. The data encompassed and compared demographic features, medical history, comorbidities, laboratory results, surgical variables and postoperative outcomes. Demographic characteristics included gender, age, body mass index (BMI), smoking history, and alcohol consumption. Medical history and comorbidities encompassed a history of bleeding, cerebral infarction, percutaneous coronary intervention (PCI), myocardial infarction, angina, heart failure (HF), diabetes, hypertension, atrial fibrillation (AF), liver dysfunction, renal dysfunction, anemia and peptic ulcer disease. Surgical variables included the number of arteriovenous grafts, duration of cardiopulmonary bypass (CPB), intraoperative blood loss, volume of intraoperative transfusion and postoperative drainage. Laboratory results consisted of diagnostic tests conducted at admission and test data collected within 48 h after surgery.

Postoperative outcomes were assessed through telephone follow-ups, face-to-face interviews and the most reliable evidence from medical records. Two independent researchers evaluated both major and minor bleeding events and they remained unaware of the patients’ bleeding risk factors during their assessments. A consensus was required for the final diagnosis, if the researchers disagreed, an independent general practitioner would make the final determination. Importantly, any bleeding events that occurred after the discontinuation of anticoagulant and antiplatelet therapy were not recorded.

### 2.4 Outcome and definition

The study outcomes include any bleeding events that occurred within 3 months after discharge. Due to the inherent differences in bleeding assessment tools, definitions of minor and major bleeding have historically varied (An et al., 2023; Okumura et al., 2020). In this study, we followed the criteria set by the International Society on Thrombosis and Haemostasis (ISTH) (Schulman et al., 2005), which defines major bleeding as: (1) fatal bleeding; (2) symptomatic bleeding in critical areas or organs, such as the brain, spinal canal, eyes, gastrointestinal tract, joints, or muscles accompanied by compartment syndrome; (3) bleeding events that result in a decrease in hemoglobin levels of at least 2 g/dL or necessitate the transfusion of at least 2 units of whole blood or red blood cells. All other bleeding events were classified as clinically relevant non-major bleeding and minor bleeding. Clinically relevant non-major bleeding was defined as bleeding that required hospitalization, medical or surgical intervention, unplanned medical attendance, or modification of antithrombotic therapy under the guidance of a physician. Other overt bleeding events that did not meet the criteria for major bleeding or clinically relevant non-major bleeding were categorized as minor bleeding, such as epistaxis or gingival bleeding. Alcohol abuse is defined as consuming ≥8 units of alcohol per week. Conditions such as chronic dialysis, kidney transplantation, or serum creatinine levels ≥200 mmol/L are considered renal dysfunction. Liver dysfunction is defined as chronic liver disease (e.g., cirrhosis) or significant biochemical evidence of liver impairment, such as bilirubin levels exceeding twice the normal upper limit, along with aspartate aminotransferase or alanine aminotransferase levels more than three times the upper limit of normal.

### 2.5 Statistical analysis

Using the random number method, we randomly divided the subjects into a training set and a validation set at a ratio of 7:3. The training set was used to develop the prediction model, while the validation set was employed to assess the model’s performance and generalization ability. Subsequently, we carefully evaluated the comparability between the two datasets. For normally distributed continuous variables, results are presented as mean ± standard deviation, with group differences assessed using Student's t-test. Non-normally distributed continuous variables are expressed as median and interquartile range (IQR), with comparisons made by the Mann-Whitney U test. Categorical variables are reported as counts (percentages) and analyzed by the chi-square test or Fisher’s exact test.

Initially, we performed univariate logistic regression to screen candidate predictive factors and selected those with *P* < 0.05 for further analysis. In the training set, we employed tenfold cross-validated Least Absolute Shrinkage and Selection Operator (LASSO) regression to identify the best predictive factors. Subsequently, multivariate logistic regression analysis was conducted by a backward stepwise approach and incorporated features identified through LASSO to determine independent predictors. After the multivariate analysis, variables with a two-sided *p*-value < 0.05 were included in the final model. Moreover, we adhered to Harrell’s guidelines during model construction, in order to ensuring a minimum event-to-covariate ratio of 10:1 (Balachandran et al., 2015). We also evaluated potential multicollinearity issues through the Variance Inflation Factor (VIF).

We evaluated discrimination capacity of the model through the area under the receiver operating characteristic curve (AUROC). The alignment between predicted probabilities of the model and actual outcomes was assessed by using calibration curves derived from 500 bootstrap resamples. Goodness of fit of this model was further examined by the Hosmer-Lemeshow test. To quantify the enhancement in predictive capability of the new model compared to the HAS-BLED scoring system, we applied net reclassification improvement (NRI) and integrated discrimination improvement (IDI) (Pencina et al., 2011; Uno et al., 2013). Additionally, we conducted decision curve analysis (DCA) to assess the net benefits at various threshold probabilities in the validation set so as to determine the clinical utility of the new model relative to the HAS-BLED scoring system (Sadatsafavi et al., 2021). Lastly, we created clinical impact curves (CIC) to provide further insight into the clinical benefits and practical applicability of the model. (See Figure 1 for the relevant technical flowchart.).

Data analysis was performed using R version 4.2.1 (R Project for Statistical Computing). The R packages utilized in this study comprised comparegroups, glm, glmnet, fbroc, rms, ResourceSelection, rmda, nricens, PredictABEL and Dcurves. All statistical tests were two-sided, with a *P* < 0.05 deemed statistically significant.

## 3 Results

### 3.1 Characteristics of patients and disease

This study enrolled 500 adult patients who underwent cardiac surgery followed by anticoagulation and antiplatelet therapy at Nanjing Drum Tower Hospital between January 2020 and June 2024 (Figure 1). The average age of participants was 68 years (IQR: 60–73), with 331 patients (66.2%) being male and 105 patients (21%) experienced bleeding events. Detailed demographic and clinical characteristics are provided in Supplementary Table S1. The patients were randomly allocated into a training set (350 patients) and a validation set (150 patients) in a 7:3 ratio. The incidence of bleeding was comparable between the two groups, with 72 patients (20.57%) experiencing bleeding events in the training set and 33 patients (22.00%) in the validation set (*p* = 0.811). Furthermore, there were no significant differences in other clinical characteristics, laboratory indicators or surgery-related variables between the two datasets (*P* > 0.05), as outlined in Supplementary Table S2.

### 3.2 Bleeding overview

The incidence of bleeding among patients in the entire study cohort was 21% (Supplementary Figure S1A). Each patient was classified into specific bleeding types based on the diagnostic criteria for major bleeding, clinically relevant non-major bleeding and minor bleeding events. The most common type was minor bleeding (76.20%), followed by clinically relevant non-major bleeding (21.90%) and major bleeding was the least common (1.90%) (Supplementary Figure S1B).

### 3.3 Model variable screening

**FIGURE 2:**
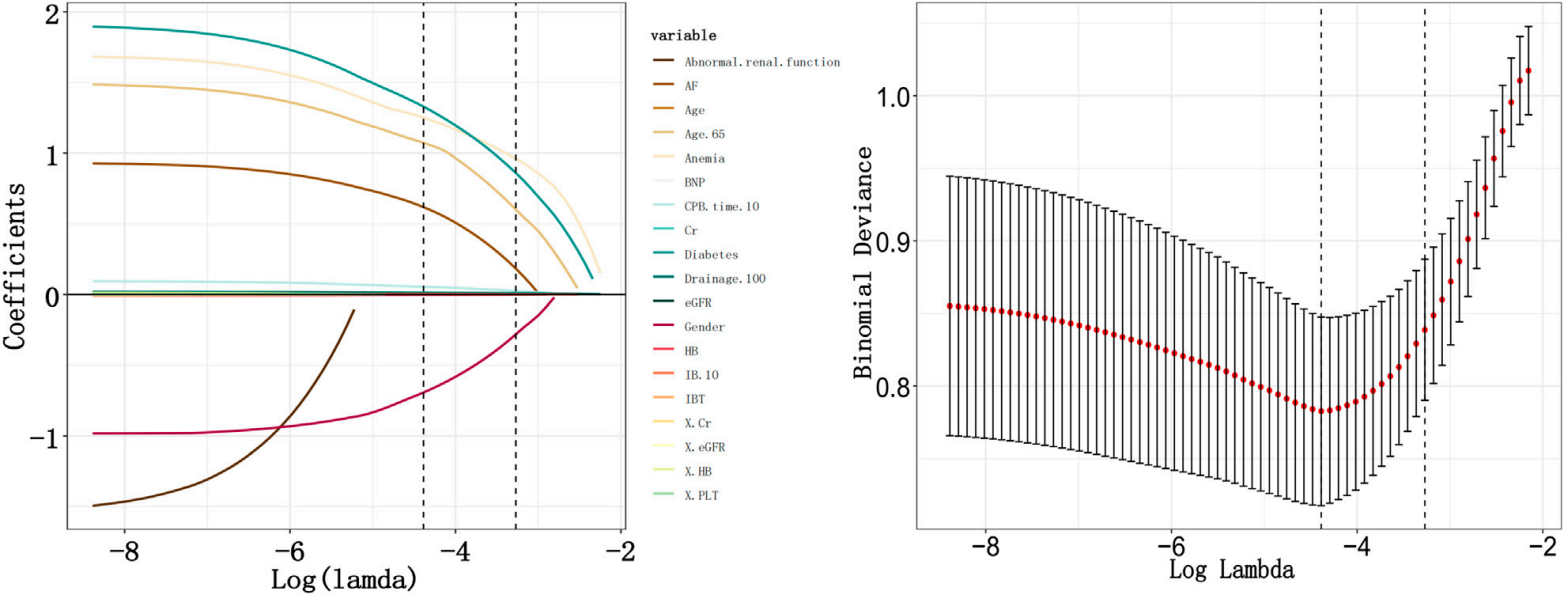
Variable selection by Least Absolute Shrinkage and Selection Operator (LASSO) regression. Drainage/100: Postoperative drainage (mL)/100; CPB time/10: cardiopulmonary bypass time (min)/10; IB/10: Intraoperative bleeding (mL)/10.

**FIGURE 3:**
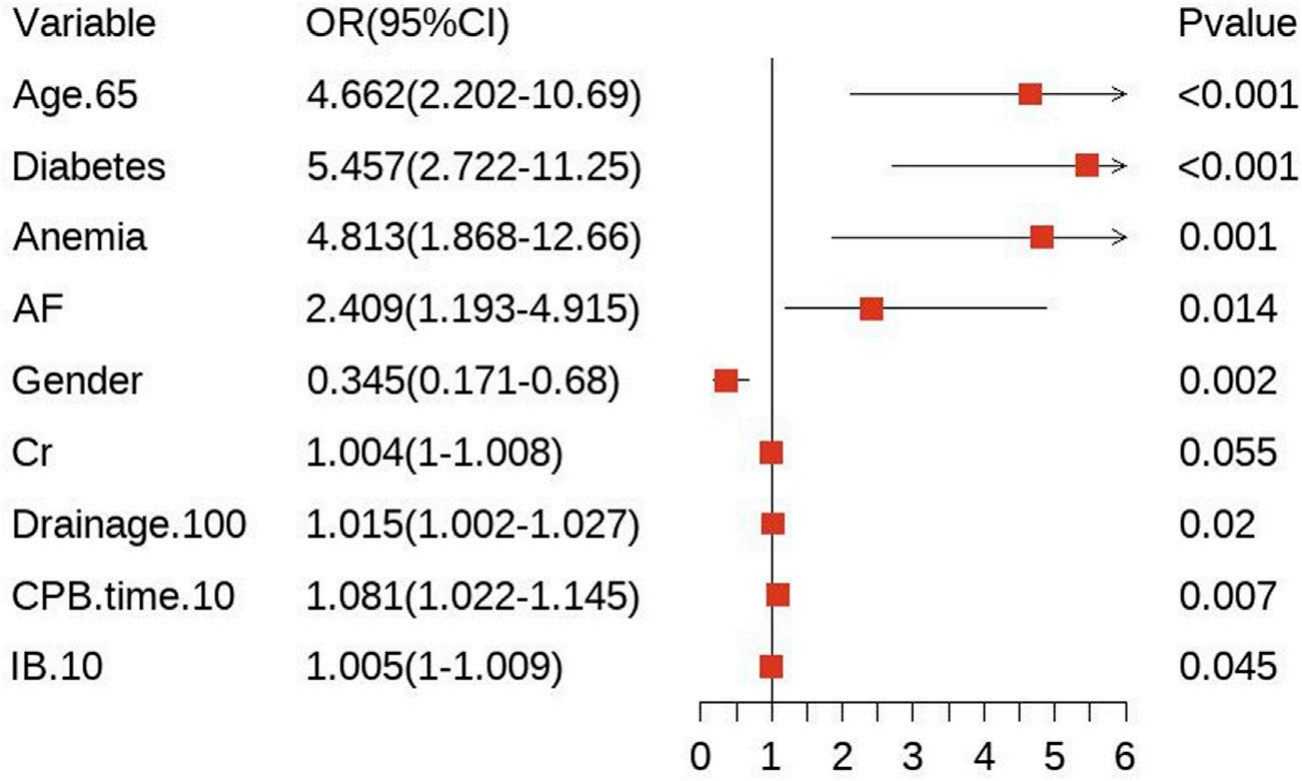
A forest plot of the multivariable analysis. Drainage/100: Postoperative drainage (mL)/100; CPB time/10: cardiopulmonary bypass time (min)/10; IB/10: Intraoperative bleeding (mL)/10.

In the training cohort, we initially conducted univariate logistic regression analysis on all predictor variables to identify those with *P* < 0.05 (Supplementary Table S3) and followed by LASSO regression analysis using ten-fold cross-validation. The analysis revealed that 10 predictor factors exhibited non-zero regression coefficients (Figure 2). These 10 variables were then included in a multivariate logistic regression analysis and independent predictors significantly associated with bleeding risk were identified using a backward stepwise approach (Supplementary Table S4). The findings from the multivariate analysis are illustrated in a forest plot (see Figure 3). Notably, age > 65 years (OR: 4.662, 95% CI: 2.202–10.69, *p* < 0.05) was significantly linked to an increased risk of bleeding. Gender (OR: 0.345, 95% CI: 0.171–0.680, *p* = 0.002) indicated that female patients faced a higher bleeding risk. Other significant predictors of bleeding included Diabetes (OR: 5.457, 95% CI: 2.722–11.25, *p* < 0.05), Anemia (OR: 4.813, 95% CI: 1.868–12.66, *p* = 0.001), AF (OR: 2.409, 95% CI: 1.193–4.915, *p* = 0.014), Drainage/100 (OR: 1.015, 95% CI: 1.002–1.027, *p* = 0.02), CPB time/10 (OR: 1.081, 95% CI: 1.022–1.145, *p* = 0.007), and IB/10 (OR: 1.005, 95% CI: 1–1.009, *p* = 0.045) (P < 0.05). Importantly, no significant multicollinearity was observed among the selected variables (Supplementary Table S4).

### 3.4 Development, validation, assessment and nomogram creation for bleeding prediction models

**FIGURE 4:**
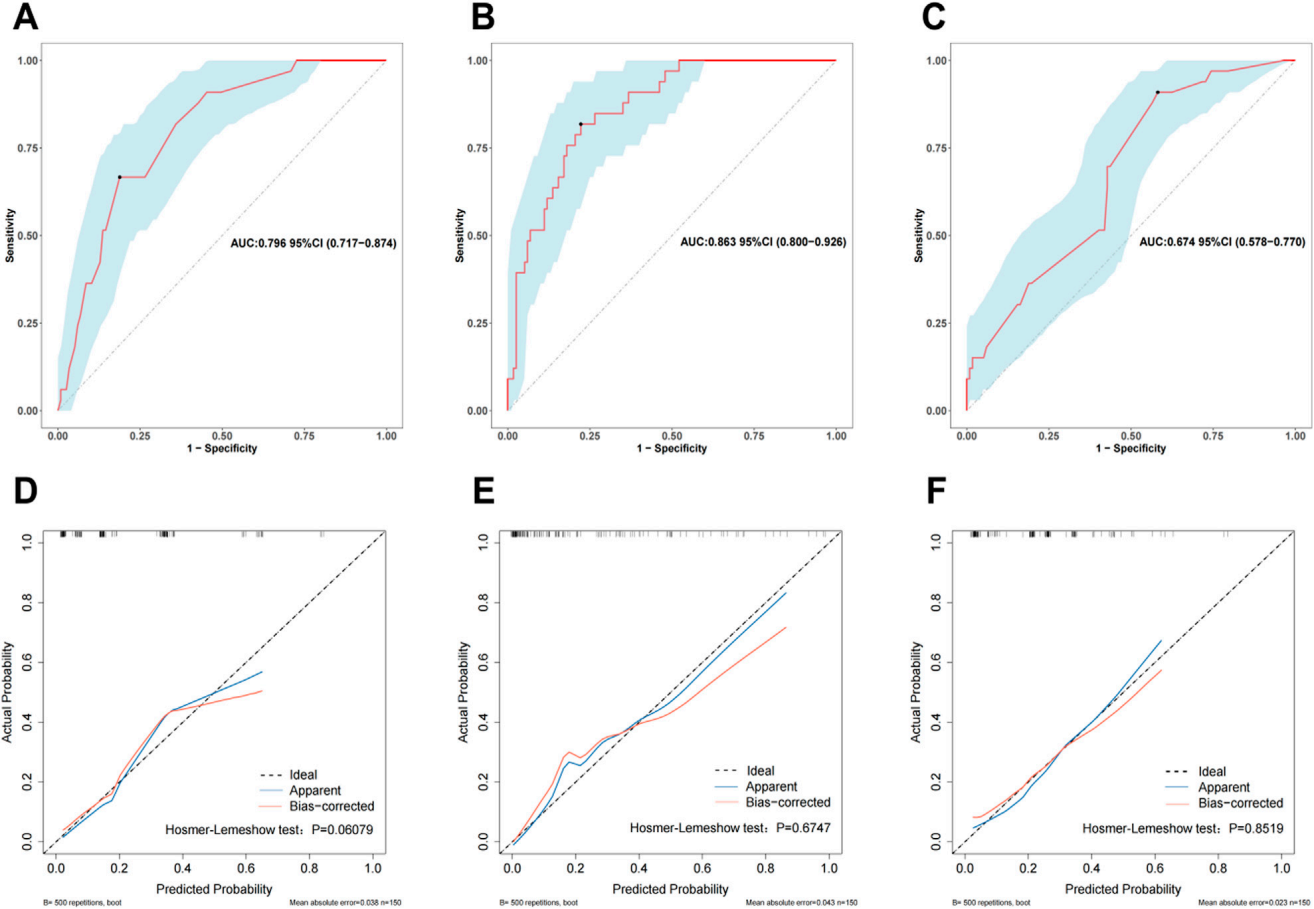
The receiver operating characteristic (ROC) curves for Mod1 **(A)**, Mod2 **(B)**, and Mod3 **(C)** in the validation cohort. Calibration curves for Mod 1 **(D)**, Mod 2 **(E)**, and Mod 3 **(F)** in the validation cohort. The ROC curve is a graphical representation that describes the performance of a binary classifier under different thresholds. The Area Under the Curve (AUC) is used to evaluate the classifier’s performance, where an AUC value closer to 1 indicates better performance. A calibration curve is employed to assess how closely the model’s predicted probabilities align with actual occurrences. If the calibration curve lies above the reference line, it suggests that the true positive probabilities are higher than the predicted probabilities, indicating underestimation of risk. Conversely, if the calibration curve lies below the reference line, it means the predicted probabilities are lower than the true positive probabilities, indicating an overestimation of risk.

While utilizing the variables identified from our multivariable analysis alongside the HAS-BLED score, we developed three bleeding risk prediction models, referred to as Mod1, Mod2 and Mod3 and assessed their performance in the validation cohort via receiver operating characteristic (ROC) curves. Mod1 incorporates preoperative factors including gender, age > 65 years, diabetes, anemia and AF—achieving an area under the curve (AUC) of 0.796 (95% CI: 0.717–0.874) as illustrated in Figure 4A. Mod2 integrates multimodal data, including preoperative characteristics, intraoperative variables and postoperative clinical features (Gender, Age > 65 years, Diabetes, Anemia, AF, CPB time, intraoperative blood loss (IB), Postoperative drainage), which achieves an AUC of 0.863 and a 95% CI of 0.800–0.926 (Figure 4B). Mod3 relies on the HAS-BLED scoring system, which includes factors such as hypertension, history of stroke, bleeding history, liver dysfunction, renal dysfunction, age > 65 years and alcohol abuse, with an AUC of 0.674 (95% CI: 0.578–0.770) depicted in Figure 4C. Calibration curves validated through 500 bootstrap resamples indicated a strong alignment between the actual occurrence of bleeding events and the predicted probabilities (refer to Figures 4D–F). The goodness-of-fit of the models was evaluated by using the Hosmer-Lemeshow test, which confirmed that all three models exhibited satisfactory fit: Mod1 (*P* = 0.061), Mod2 (*P* = 0.675) and Mod3 (*P* = 0.852).

**FIGURE 5:**
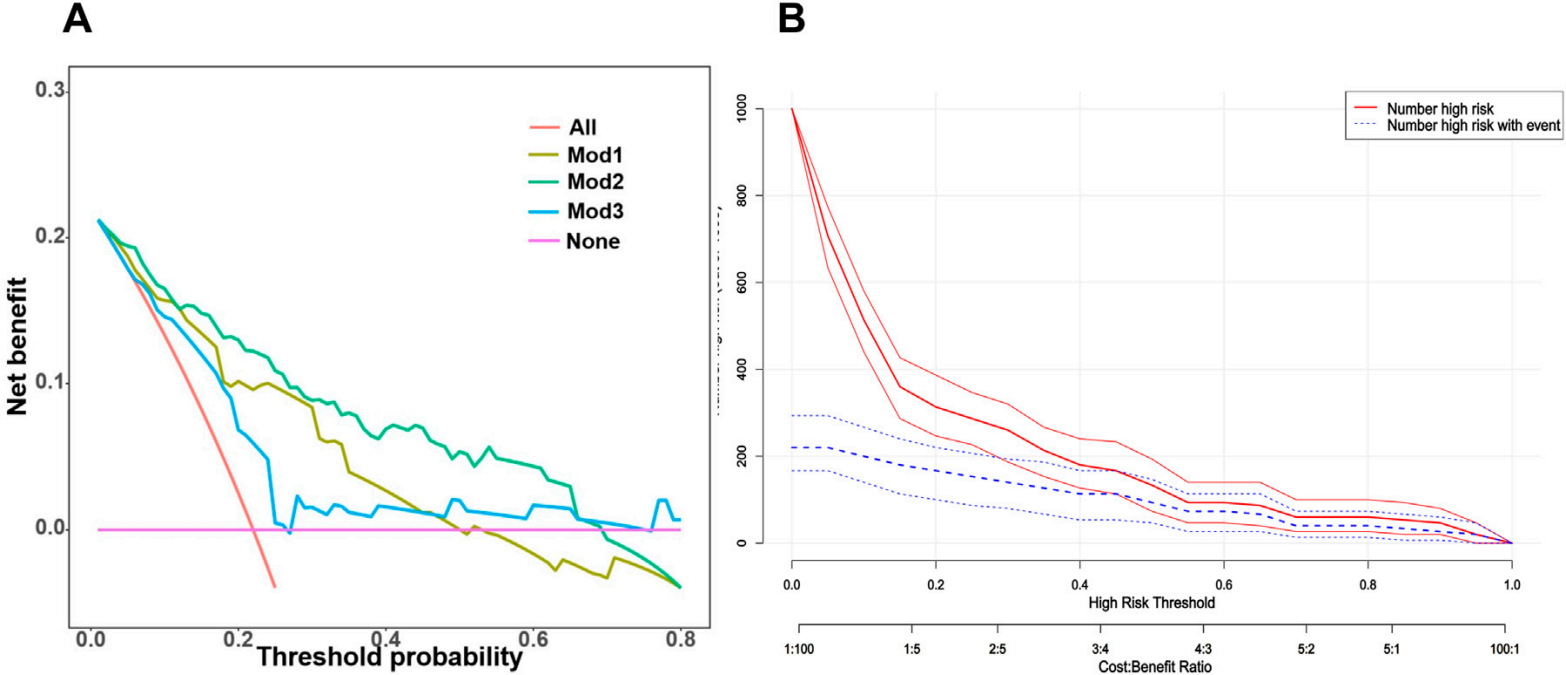
**(A)** A summary plot of the Decision Curve Analysis (DCA) curves for the three bleeding risk prediction models in the validation cohort. The horizontal axis represents the threshold probability, which is the cutoff used to determine whether a patient should receive treatment. If the model’s predicted probability exceeds this threshold, the patient is classified as a “positive case” and should receive treatment. Conversely, if the predicted probability is below the threshold, the patient is defined as a “negative case” and does not receive treatment. **(B)** The Clinical Impact Curve (CIC) of Mod2 in the validation cohort. The clinical impact curve graphically illustrates the expected number of individuals who will be correctly or incorrectly diagnosed, treated, or classified under different threshold or decision points. This visual representation aids doctors, researchers and policymakers in understanding the potential impact of specific medical interventions. Primarily focusing on the true positive rate (sensitivity) and false positive rate (1-specificity), the clinical impact curve measures the predictive model’s ability to accurately identify patients with bleeding and its tendency to mistakenly label healthy individuals as bleeding patients.

**FIGURE 6:**
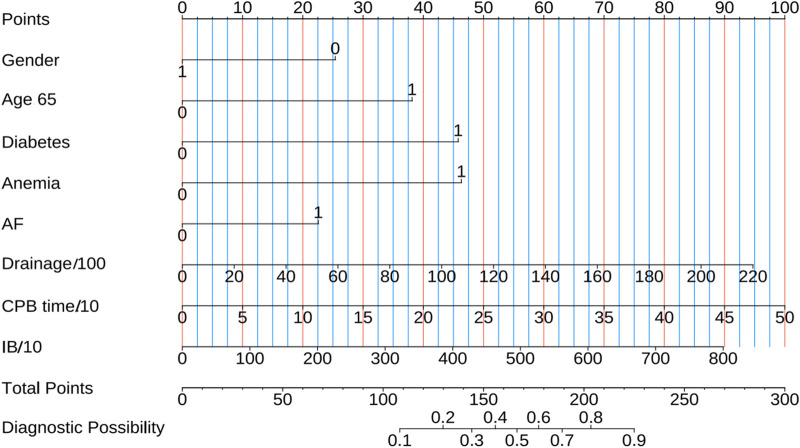
The nomogram predicts short-term bleeding risk in patients undergoing combined anticoagulant and antiplatelet therapy following cardiac surgery. Gender: 0 for female and 1 for male; Age 65:1 means Age > 65 years; Diabetes, Anemia and AF: 0 means No and 1 indicates Yes; Drainage/100: Postoperative drainage (mL)/100; CPB time/10: cardiopulmonary bypass time (min)/10; IB/10: Intraoperative bleeding (mL)/10.

We further assessed the enhancement in bleeding risk prediction performance of Mod2 compared to Mod1 and Mod3 compared to Mod2 with NRI and IDI metrics (Table 1). The analysis revealed that Mod2 achieved an NRI of 0.160 (95% CI: 0.032–0.288, *P* = 0.014) and an IDI of 0.151 (95% CI: 0.088–0.214, *P* < 0.001) compared to Mod1. In comparison to Mod3, Mod2 had an NRI of 0.217 (95% CI: 0.009–0.425, *P* = 0.041) and an IDI of 0.194 (95% CI: 0.079–0.308, *P* < 0.001). These findings suggest that Mod2 offers greater accuracy in bleeding risk prediction than both Mod1 and Mod3. Additionally, we evaluated the clinical utility of each model through DCA and presented the comparisons (Figure 5A). The results indicated that Mod2 provided the highest net benefit across threshold probabilities of 2%–70%, which implyed that its use within this range is more advantageous than the default approaches of treating all patients or none. However, the net benefit of Mod2 was limited at threshold probabilities below 2% or above 70%. The CIC further supported that Mod2 exhibits robust risk prediction capabilities and significant clinical relevance (Figure 5B). Considering the findings from various assessment methods, Mod2 showed the best performance, so we selected it for developing a nomogram applicable in clinical practice (Figure 6).

**TABLE 1:** The Net Reclassification Improvement (NRI) and Integrated Discrimination Improvement (IDI) based on Mod1, Mod2, and Mod3. The Net Reclassification Improvement (NRI) and the Integrated Discrimination Improvement (IDI) are metrics used to evaluate the performance enhancement of a new predictive model compared to an existing one. By calculating these indices, we can objectively assess the degree of improvement offered by the new model relative to the old model.

Index	Training cohort		Validation cohort	
	95%CI	*P*-value	95%CI	*P*-value
NRI				
Model 1 vs. Model 2	0.118 (0.026–0.211)	**0.012**	0.160 (0.032–0.288)	**0.014**
Model 3 vs. Model 2	0.302 (0.187–0.417)	**<0.001**	0.217 (0.009–0.425)	**0.041**
IDI				
Model 1 vs. Model 2	0.099 (0.063–0.135)	**<0.001**	0.151 (0.088–0.214)	**<0.001**
Model 3 vs. Model 2	0.238 (0.172–0.304)	**<0.001**	0.194 (0.079–0.308)	**<0.001**

## 4 Discussion

This study successfully developed and validated a novel nomogram that incorporates eight significant risk factors: gender, age, diabetes, anemia, AF, CPB time, intraoperative blood loss and postoperative drainage. This nomogram is designed to predict the short-term bleeding risk for patients receiving anticoagulation combined with antiplatelet therapy following cardiac surgery. When compared to the traditional HAS-BLED scoring model and a model that only considered preoperative characteristics, the nomogram showed positive values for both the NRI and IDI (*P* < 0.05) and demonstrated its superior predictive capability over the HAS-BLED model. Additionally, DCA confirmed that the nomogram provides enhanced clinical utility and practicality in assessing the bleeding risk for patients.

The limitations of the HAS-BLED score in specific patient populations deserve further exploration (Gulec, 2014). Although it performs well in general bleeding risk assessment, the HAS-BLED score relies primarily on a few preoperative clinical parameters, such as hepatic insufficiency and alcohol intake (Lip et al., 2011), which may not adequately reflect the complexity of bleeding risk in certain high-risk groups. Furthermore, the exclusion of intraoperative and postoperative variables in the HAS-BLED score limits its applicability to patients receiving antithrombotic therapy after cardiac surgery.

To overcome these limitations, our nomogram model innovatively incorporates intraoperative and postoperative variables, including cardiopulmonary bypass time, intraoperative blood loss, and postoperative drainage volume. These factors enable a more comprehensive capture of dynamic changes that affect bleeding risk. Systematic evaluation of model performance, including AUC, NRI, and IDI metrics, demonstrates that the nomogram not only improves predictive accuracy but also significantly enhances the applicability of clinical decision-making. The results of decision curve analysis further confirm the potential value of the nomogram in guiding personalized treatment strategies.

Additionally, the nomogram illustrates the complex interactions of preoperative, intraoperative, and postoperative variables, emphasizing the importance of multidimensional risk assessment in antithrombotic therapy. Especially in patients at high risk of bleeding, predictions based on the nomogram can provide clinicians with more accurate risk assessments, facilitating the development of personalized intervention strategies to optimize patient outcomes. By integrating more comprehensive clinical data, our model offers a more reliable tool for bleeding risk assessment in future clinical practice and provides a valuable reference framework for related research.

Artificial Intelligence (AI) displayed great potential in medical informatics and disease management (Lin, 2024), including the Cardiovascular Disease (Sahrawat et al., 2024). Moreover, AI-based nomogram could be used to predict the prognostic values (Guo et al., 2024). Studies indicate that AI-based nomograms offer substantial advantages over traditional scoring systems in the context of clinical prediction models. Firstly, nomograms explained graphically how different predictive factors influence clinical outcomes, which enabled physicians to quickly grasp the combined effects of various factors. This visual approach enhances information transparency and minimizes the risk of calculation errors. Secondly, nomograms effectively integrate complex data, which leads to more accurate prognostic evaluations. However traditional scoring systems may introduce biases due to simplification, which ultimately contributes to prediction accuracy reduction. As a result, in contemporary clinical practice, nomograms not only enhance the efficiency of decision-making but also serve as a more reliable and user-friendly tool for physicians (Balachandran et al., 2015; Park, 2018). This further validates the findings of this study and highlights their importance in clinical practice.

Previous studies have highlighted considerable differences in bleeding predictive factors across various surgical types and the use of different antithrombotic agents. Nevertheless, gender and age are consistently recognized as independent risk factors (Fortuni et al., 2018; Forbes et al., 2024; Alfredsson et al., 2017), which is consistent with our results. They often experience vascular degeneration and various comorbidities, which may account for the heightened bleeding risk in older patients (Liu et al., 2020). A large cohort study conducted by Gregory and colleagues on 7,329 patients with atrial fibrillation using oral anticoagulants to prevent stroke found that diabetes was identified as an independent risk factor for bleeding through multivariable analysis (HR: 1.47; 95% CI: 1.10–1.97; *p* = 0.009) (Lip et al., 2011). We hypothesize that elevated blood glucose levels can compromise the integrity and repair mechanisms of blood vessel walls, which makes them more susceptible to damage. Additionally, chronic hyperglycemia may lead to microvascular complications, which increases the risk of localized bleeding. However, the potential pathophysiological mechanisms behind this may be multifaceted.

Our findings further support anemia as an independent predictor of bleeding. In a prospective multicenter cohort study, Zenati et al. identified anemia as a key independent risk factor for major bleeding in patients receiving long-term VKA therapy (Zenati et al., 2017). Low hemoglobin levels can contribute to coagulation disorders, impaired hemostasis and extended bleeding times (Li et al., 2022; Roh et al., 2019). Moreover, reduced hemoglobin levels may indicate the presence of comorbidities and overall patient frailty, which can significantly elevate bleeding risks during combined anticoagulant with antiplatelet therapy (Zenati et al., 2017). Consistent with previous reports, patients with baseline anemia face a substantially higher risk of bleeding events compared to non-anemic patients (Zenati et al., 2017; Faggioni et al., 2019; Kunadian et al., 2014).

Huang et al.’s research identified prolonged CPB time as an independent risk factor for post-cardiac surgery headaches (OR: 1.019, 95%CI: 1.017–1.020, *P* < 0.001), with longer CPB times generally indicating poorer clinical outcomes (Wang et al., 2022). This finding lends indirect support to the rationale behind our results, though the precise mechanisms remain to be elucidated.

It is also unsurprising that intraoperative blood loss is closely linked to bleeding events in patients receiving postoperative antithrombotic therapy. In patients on combined anticoagulant with antiplatelet regimens following cardiac surgery, these drugs inhibit coagulation factor activity and platelet aggregation, which heightens the bleeding risk. Excessive intraoperative blood loss depletes clotting factors and platelets and further amplifys this risk. Additionally, greater blood loss during surgery typically necessitates more blood transfusions. Although red blood cell transfusions are a common and lifesaving intervention in cardiac surgery, they have been associated with adverse outcomes (Doyle et al., 2009; Sultan et al., 2023). The potential pathophysiological mechanisms are likely multifaceted. Furthermore, red blood cell distribution width often increases after transfusion, which may impair microcirculation due to decreased red blood cell deformability, thus the likelihood of bleeding events increases (Doyle et al., 2009; Lu et al., 2024).

In summary, the nomogram significantly contributes to individualized risk assessment, identifying high-risk patients and guiding intervention strategies. Based on this, we believe that individualized treatment strategies should become an essential component of managing high-risk patients. By accurately identifying patients at high risk of postoperative bleeding, we can take more refined intervention measures in clinical decision-making, thereby effectively reducing bleeding events and improving patient outcomes. For instance, in the preoperative stage, optimized interventions for high-risk patients, such as correcting anemia and controlling hyperglycemia, can not only reduce the risk of postoperative bleeding but also improve the overall surgical tolerance and postoperative recovery ability of patients. These interventions can assist doctors in better tailoring treatment plans for patients and reducing the incidence of complications.

During surgery, controlling cardiopulmonary bypass time and reducing intraoperative blood loss are key factors in lowering the risk of postoperative bleeding. These two factors largely reflect the surgeon’s experience and surgical skills. Undoubtedly, experienced surgeons require shorter operative times and less intraoperative blood transfusion under the same conditions. Therefore, enhancing the surgeon’s operational experience and proficiency may be an effective strategy to reduce bleeding and other adverse events.

In the postoperative stage, we recommend implementing a graded management system. Clinical pharmacists can provide personalized anticoagulation therapy guidance for high-risk patients based on their bleeding risk stratification. They can adjust medication dosages for patients according to our proposed model, reducing unnecessary over-anticoagulation treatment and thereby decreasing the risk of bleeding. Additionally, pharmacists can conduct additional follow-ups for high-risk patients, regularly monitoring their bleeding manifestations to ensure early detection of potential bleeding events and take intervention measures. This personalized management and follow-up strategy can help reduce postoperative bleeding and bring more net benefits.

Our study has several noteworthy strengths. To the best of our knowledge, this is the first model specifically designed to predict short-term bleeding risk in patients undergoing combined anticoagulation with antiplatelet therapy after cardiac surgery. We utilized the lasso method to select variables with a *p*-value < 0.05 in univariate analysis, followed by 10-fold cross-validation to regularize regression coefficients, which improved prediction accuracy and preventing overfitting. Furthermore, we innovatively compared the HAS-BLED score with two newly developed models and performed a comprehensive evaluation by multiple indicators such as AUC, calibration curves, Hosmer-Lemeshow test, DCA, NRI, IDI and CIC. Based on these evaluations, we selected the best-performing model and constructed a user-friendly nomogram. This nomogram integrates preoperative, intraoperative and postoperative variables, which enables clinicians to predict bleeding risks more accurately. Additionally, the model highlights the intricate interactions between variables across different stages of care (preoperative, intraoperative, postoperative), and underscores many dimensions of bleeding risk assessment in antithrombotic therapy. The incorporation of multiple data types further enhances the precision of clinical predictions.

However, our study is not without limitations. Firstly, the nomogram was derived from retrospective data collected between 2020 and 2024 and the loss of follow-up reduced the effective sample size, which potentially affected internal validity. Secondly, due to the observational study, residual confounding factors related to bleeding risk cannot be entirely excluded (Donzé et al., 2013). Furthermore, during the random splitting of the two datasets, there may be potential unavoidable biases. Lastly, this was a single-center retrospective study and the nomogram was only validated internally. Its generalizability requires further confirmation through external validation in multi-center studies.

## 5 Conclusion

In summary, based on real-world data, we identified 8 independent bleeding risk factors and developed a simple yet robust nomogram for personalized evaluation of bleeding risk within 3 months after discharge in patients undergoing combined anticoagulant with antiplatelet therapy following cardiac surgery. This nomogram outperforms the widely used HAS-BLED scoring model and models that solely consider preoperative characteristics in predictive accuracy, discrimination and clinical applicability, thus it can enhance the management, monitoring and improve outcomes of patients. Additionally, the nomogram, including factors relevant to the intraoperative and postoperative phases, may offer new insights for future research.

## Data Availability

The original contributions presented in the study are included in the article/Supplementary Material, further inquiries can be directed to the corresponding authors.
