# Supplementary material for: Doctor, what is my risk of bleeding after cardiac surgery while on combined anticoagulant with antiplatelet therapy? A validated nomogram for risk assessment
Source: Front Pharmacol. 2025 Jan 7;15:1528390. doi: 10.3389/fphar.2024.1528390 (PMC11747104; doi:10.3389/fphar.2024.1528390)
Supplement: Supplementary file 1 [file DataSheet1.docx]

Supplementary Material

**Table S1. Demographic characteristics, medical history, laboratory test results, surgical and postoperative variables of patients with and without bleeding.**

| **Characteristics** | **Total N=500** | **No Bleeding N=395** | **Bleeding N=105** | **P value** |
| --- | --- | --- | --- | --- |
| Gender, n(%) |  |  |  | 0.005 |
| Female | 169 (33.80%) | 121 (30.63%) | 48 (45.71%) |  |
| Male | 331 (66.20%) | 274 (69.37%) | 57 (54.29%) |  |
| Age (years), median (Q1, Q3) | 68.00 [60.00;73.00] | 67.00 [59.00;72.50] | 70.00 [66.00;75.00] | <0.001 |
| Age>65 (years), n (%) | 298 (59.60%) | 211 (53.42%) | 87 (82.86%) | <0.001 |
| BMI (kg/m^2^)>25, n (%) | 23.90 [21.48;26.22] | 24.00 [21.50;26.40] | 23.50 [21.20;26.00] | 0.261 |
| Smoking, n (%) | 109 (21.80%) | 85 (21.52%) | 24 (22.86%) | 0.871 |
| Drinking≥8U/wk, n (%) | 69 (13.80%) | 55 (13.92%) | 14 (13.33%) | 1 |
| Hypertension, n (%) | 316 (63.20%) | 241 (61.01%) | 75 (71.43%) | 0.064 |
| Diabetes, n (%) | 110 (22.00%) | 66 (16.71%) | 44 (41.90%) | <0.001 |
| Abnormal liver function, n (%) | 66 (13.20%) | 50 (12.66%) | 16 (15.24%) | 0.595 |
| Abnormal renal function, n (%) | 18 (3.60%) | 9 (2.28%) | 9 (8.57%) | 0.005 |
| Angina pectoris, n (%) | 58 (11.60%) | 44 (11.14%) | 14 (13.33%) | 0.651 |
| Myocardial infarction, n (%) | 59 (11.80%) | 45 (11.39%) | 14 (13.33%) | 0.706 |
| Heart failure, n (%) | 20 (4.00%) | 13 (3.29%) | 7 (6.67%) | 0.156 |
| PCI, n (%) | 50 (10.00%) | 39 (9.87%) | 11 (10.48%) | 1 |
| Anemia, n (%) | 61 (12.20%) | 32 (8.10%) | 29 (27.62%) | <0.001 |
| Previous bleeding, n (%) | 10 (2.00%) | 5 (1.27%) | 5 (4.76%) | 0.038 |
| Stroke, n (%) | 98 (19.60%) | 68 (17.22%) | 30 (28.57%) | 0.014 |
| DVT, n (%) | 5 (1.00%) | 2 (0.51%) | 3 (2.86%) | 0.065 |
| Atrial fibrillation, n (%) | 151 (30.20%) | 102 (25.82%) | 49 (46.67%) | <0.001 |
| Peptic ulcer, n (%) | 15 (3.00%) | 9 (2.28%) | 6 (5.71%) | 0.099 |
| NYHA Classification Ⅲ-Ⅳ, n (%) | 262 (52.40%) | 211 (53.42%) | 51 (48.57%) | 0.439 |
| LVEF (%), median (Q1, Q3) | 54.00 [45.00;58.00] | 54.00 [45.05;58.00] | 53.50 [42.00;58.00] | 0.498 |
| PLT (×10^9^/L),median (Q1, Q3) | 176.00 [140.75;214.25] | 178.00 [141.00;213.50] | 167.00 [139.00;215.00] | 0.301 |
| HB (g/L), median (Q1, Q3) | 133.00 [120.00;146.00] | 135.00 [122.00;147.00] | 128.00 [112.00;139.00] | <0.001 |
| WBC (×10^9^/L), median (Q1, Q3) | 6.10 [5.10;7.32] | 6.20 [5.15;7.30] | 5.90 [5.00;7.40] | 0.641 |
| Cr (umol/L), median (Q1, Q3) | 71.00 [60.00;87.00] | 70.00 [60.00;85.00] | 73.00 [60.00;94.00] | 0.167 |
| eGFR (ml/min/1.73m^2^), mean ± SD | 96.25 [76.95;113.78] | 97.30 [78.70;114.30] | 90.80 [64.90;109.40] | 0.007 |
| ALT (U/L), median (Q1, Q3) | 18.65 [13.78;30.20] | 19.00 [13.90;31.00] | 17.50 [13.30;28.60] | 0.169 |
| AST (U/L), median (Q1, Q3) | 20.10 [16.58;28.02] | 19.90 [16.35;28.10] | 21.00 [17.30;27.50] | 0.529 |
| TBIL (umol/L), median (Q1, Q3) | 11.80 [8.30;16.52] | 11.90 [8.60;16.80] | 10.80 [7.90;15.30] | 0.111 |
| BNP (pg/mL), median (Q1, Q3) | 219.00 [77.02;519.50] | 210.75 [74.38;517.50] | 250.00 [91.60;546.00] | 0.125 |
| PT (s), median (Q1, Q3) | 11.50 [11.00;12.30] | 11.50 [10.90;12.30] | 11.60 [11.20;12.50] | 0.052 |
| APTT (s), median (Q1, Q3) | 27.80 [26.20;29.80] | 27.70 [26.20;29.70] | 27.80 [25.90;30.30] | 0.926 |
| TT (s), median (Q1, Q3) | 18.10 [17.30;19.00] | 18.10 [17.40;19.00] | 17.90 [17.30;19.00] | 0.493 |
| FIB (g/L), median (Q1, Q3) | 2.80 [2.40;3.50] | 2.80 [2.40;3.40] | 2.90 [2.40;3.50] | 0.283 |
| AKI, n (%) | 63 (12.60%) | 45 (11.39%) | 18 (17.14%) | 0.158 |
| PLT* (×10^9^/L),median (Q1, Q3) | 101.00 [79.75;128.00] | 103.00 [81.00;129.00] | 91.00 [69.00;120.00] | 0.008 |
| HB* (g/L), mean ± SD | 102.63 (15.04) | 103.98 (14.48) | 97.55 (16.05) | <0.001 |
| WBC* (×10^9^/L),median (Q1, Q3) | 11.30 [9.50;13.80] | 11.40 [9.60;13.90] | 11.30 [9.20;13.60] | 0.498 |
| Cr* (umol/L), median (Q1, Q3) | 73.00 [60.00;96.00] | 72.00 [60.00;91.50] | 79.00 [64.00;111.00] | 0.028 |
| eGFR* (ml/min/1.73m^2^), mean ± SD | 90.28 (33.51) | 92.78 (33.02) | 80.87 (33.79) | 0.002 |
| ALT* (U/L), median (Q1, Q3) | 21.55 [15.47;34.65] | 21.90 [15.90;36.15] | 18.70 [14.10;28.30] | 0.03 |
| AST* (U/L), median (Q1, Q3) | 48.85 [34.50;74.25] | 48.70 [34.55;74.00] | 50.00 [34.50;76.10] | 0.823 |
| TBIL* (umol/L), median (Q1, Q3) | 19.10 [12.70;29.02] | 19.00 [12.65;28.50] | 19.40 [13.70;33.50] | 0.352 |
| PT* (s), median (Q1, Q3) | 13.60 [12.88;14.53] | 13.60 [12.80;14.50] | 13.70 [13.00;14.70] | 0.086 |
| APTT* (s), median (Q1, Q3) | 32.55 [29.50;36.00] | 32.50 [29.40;35.70] | 33.20 [30.00;36.90] | 0.083 |
| TT* (s), median (Q1, Q3) | 17.30 [16.40;19.20] | 17.20 [16.30;18.70] | 17.90 [16.60;20.20] | 0.011 |
| FIB* (g/L), median (Q1, Q3) | 3.00 [2.50;3.50] | 3.00 [2.60;3.55] | 2.90 [2.30;3.50] | 0.071 |
| Drainage (ml)/100, median (Q1, Q3) | 16.70 [9.80;29.13] | 14.20 [8.75;23.65] | 29.70 [19.30;50.50] | <0.001 |
| Bridging number, median (Q1, Q3) | 1.00 [1.00;2.00] | 1.00 [1.00;2.00] | 1.00 [1.00;3.00] | 0.114 |
| Number of arterial grafts, n (%) |  |  |  | 0.661 |
| 0 | 149 (29.80%) | 121 (30.63%) | 28 (26.67%) |  |
| 1 | 320 (64.00%) | 251 (63.54%) | 69 (65.71%) |  |
| 2 | 29 (5.80%) | 21 (5.32%) | 8 (7.62%) |  |
| 3 | 2 (0.40%) | 2 (0.51%) | 0 (0.00%) |  |
| Number of vein grafts, n (%) |  |  |  | 0.194 |
| 0 | 201 (40.20%) | 167 (42.28%) | 34 (32.38%) |  |
| 1 | 251 (50.20%) | 189 (47.85%) | 62 (59.05%) |  |
| 2 | 47 (9.40%) | 38 (9.62%) | 9 (8.57%) |  |
| 3 | 1 (0.20%) | 1 (0.25%) | 0 (0.00%) |  |
| Duration of surgery (min), median (Q1, Q3) | 365.00 [315.00;425.00] | 360.00 [305.00;420.00] | 380.00 [330.00;440.00] | 0.006 |
| CPB time (min)/10, median (Q1, Q3) | 17.60 [14.45;21.83] | 16.90 [13.85;20.05] | 21.40 [17.10;25.00] | <0.001 |
| Ascending aortic block time (min), median (Q1, Q3) | 120.00 [90.00;150.00] | 117.00 [89.00;145.00] | 127.00 [97.00;171.00] | 0.05 |
| IB (ml)/10, median (Q1, Q3) | 100.00 [80.00;150.00] | 100.00 [80.00;140.00] | 150.00 [110.00;180.00] | <0.001 |
| IBT (ml), median (Q1, Q3) | 800.00 [0.00;1300.00] | 700.00 [0.00;1200.00] | 1175.00 [650.00;1650.00] | <0.001 |

* = Postoperative laboratory indicators

BMI=body mass index, PCI=percutaneous coronary intervention, DVT=deep vein thrombosis, NYHA=New York Heart Association, LVEF=left ventricular ejection fraction, PLT=platelets, HB=hemoglobin, WBC=white blood cells, eGFR=estimated glomerular filtration rates, ALT=Alanine amio transferase, AST=Aspartate transaminase, TBIL=total bilirubin, BNP=B-type natriuretic peptide, PT=prothrombin time, APTT=activated partial thromboplastin time, TT=thrombin time, FIB=fibrinogen, Drainage/100= Postoperative drainage(ml)/100, CPB time/10= cardiopulmonary bypass time (min)/10, IB/10= Intraoperative bleeding (ml)/10, IBT= intraoperative blood transfusion

**Table S2. A comparison of the demographic and clinical characteristics between all patients, as well as those in the training and validation cohorts.**

| **Characteristics** | **Total N=500** | **Train N=350** | **Test N=150** | **P value** |
| --- | --- | --- | --- | --- |
| Bleeding | 105 (21.00%) | 72 (20.57%) | 33 (22.00%) | 0.811 |
| Gender, n(%) |  |  |  | 0.804 |
| Female | 169 (33.80%) | 120 (34.29%) | 49 (32.67%) |  |
| Male | 331 (66.20%) | 230 (65.71%) | 101 (67.33%) |  |
| Age (years), median (Q1, Q3) | 68.00 [60.00;73.00] | 68.00 [60.00;73.00] | 68.00 [60.25;73.00] | 0.525 |
| Age>65 (years), n (%) | 298 (59.60%) | 205 (58.57%) | 93 (62.00%) | 0.538 |
| BMI (kg/m^2^)>25, n (%) | 23.90 [21.48;26.22] | 23.90 [21.33;26.10] | 23.75 [21.52;26.65] | 0.754 |
| Smoking, n (%) | 109 (21.80%) | 74 (21.14%) | 35 (23.33%) | 0.671 |
| Drinking≥8U/wk, n (%) | 69 (13.80%) | 44 (12.57%) | 25 (16.67%) | 0.282 |
| Hypertension, n (%) | 316 (63.20%) | 232 (66.29%) | 84 (56.00%) | 0.037 |
| Diabetes, n (%) | 110 (22.00%) | 80 (22.86%) | 30 (20.00%) | 0.556 |
| Abnormal liver function, n (%) | 66 (13.20%) | 48 (13.71%) | 18 (12.00%) | 0.708 |
| Abnormal renal function, n (%) | 18 (3.60%) | 10 (2.86%) | 8 (5.33%) | 0.271 |
| Angina pectoris, n (%) | 58 (11.60%) | 37 (10.57%) | 21 (14.00%) | 0.345 |
| Myocardial infarction, n (%) | 59 (11.80%) | 42 (12.00%) | 17 (11.33%) | 0.952 |
| Heart failure, n (%) | 20 (4.00%) | 13 (3.71%) | 7 (4.67%) | 0.803 |
| PCI, n (%) | 50 (10.00%) | 35 (10.00%) | 15 (10.00%) | 1 |
| Anemia, n (%) | 61 (12.20%) | 37 (10.57%) | 24 (16.00%) | 0.121 |
| Previous bleeding, n (%) | 10 (2.00%) | 6 (1.71%) | 4 (2.67%) | 0.496 |
| Stroke, n (%) | 98 (19.60%) | 66 (18.86%) | 32 (21.33%) | 0.606 |
| DVT, n (%) | 5 (1.00%) | 3 (0.86%) | 2 (1.33%) | 0.639 |
| Atrial fibrillation, n (%) | 151 (30.20%) | 100 (28.57%) | 51 (34.00%) | 0.269 |
| Peptic ulcer, n (%) | 15 (3.00%) | 9 (2.57%) | 6 (4.00%) | 0.399 |
| NYHA Classification Ⅲ-Ⅳ, n (%) | 262 (52.40%) | 178 (50.86%) | 84 (56.00%) | 0.338 |
| LVEF (%), median (Q1, Q3) | 54.00 [45.00;58.00] | 54.00 [45.00;58.00] | 54.00 [44.25;57.98] | 0.699 |
| PLT (×10^9^/L),median (Q1, Q3) | 176.00 [140.75;214.25] | 176.00 [143.25;210.00] | 176.50 [131.00;227.50] | 0.621 |
| HB (g/L), median (Q1, Q3) | 133.00 [120.00;146.00] | 133.00 [119.25;147.00] | 132.50 [120.00;142.00] | 0.444 |
| WBC (×10^9^/L), median (Q1, Q3) | 6.10 [5.10;7.32] | 6.25 [5.20;7.50] | 5.90 [5.00;7.10] | 0.084 |
| Cr (umol/L), median (Q1, Q3) | 71.00 [60.00;87.00] | 71.00 [62.00;86.00] | 70.50 [59.00;88.00] | 0.552 |
| eGFR (ml/min/1.73m^2^), mean ± SD | 96.25 [76.95;113.78] | 95.25 [76.20;113.07] | 98.00 [77.62;116.07] | 0.227 |
| ALT (U/L), median (Q1, Q3) | 18.65 [13.78;30.20] | 18.95 [13.33;31.30] | 17.90 [14.43;28.90] | 0.911 |
| AST (U/L), median (Q1, Q3) | 20.10 [16.58;28.02] | 19.95 [16.62;28.48] | 20.30 [16.33;26.50] | 0.783 |
| TBIL (umol/L), median (Q1, Q3) | 11.80 [8.30;16.52] | 11.90 [8.60;16.40] | 11.60 [8.03;16.90] | 0.725 |
| BNP (pg/mL), median (Q1, Q3) | 219.00 [77.02;519.50] | 217.20 [73.96;509.34] | 220.00 [82.62;550.50] | 0.818 |
| PT (s), median (Q1, Q3) | 11.50 [11.00;12.30] | 11.55 [11.00;12.30] | 11.50 [10.90;12.50] | 0.399 |
| APTT (s), median (Q1, Q3) | 27.80 [26.20;29.80] | 27.70 [26.00;29.80] | 27.80 [26.40;29.80] | 0.366 |
| TT (s), median (Q1, Q3) | 18.10 [17.30;19.00] | 18.10 [17.30;19.10] | 18.00 [17.40;18.78] | 0.42 |
| FIB (g/L), median (Q1, Q3) | 2.80 [2.40;3.50] | 2.80 [2.40;3.40] | 2.90 [2.40;3.68] | 0.145 |
| AKI, n (%) | 63 (12.60%) | 46 (13.14%) | 17 (11.33%) | 0.681 |
| PLT* (×10^9^/L),median (Q1, Q3) | 101.00 [79.75;128.00] | 103.00 [79.00;126.00] | 99.00 [80.00;130.50] | 0.904 |
| HB* (g/L), mean ± SD | 102.63 (15.04) | 102.65 (14.75) | 102.59 (15.75) | 0.969 |
| WBC* (×10^9^/L),median (Q1, Q3) | 11.30 [9.50;13.80] | 11.35 [9.50;14.10] | 11.20 [9.40;13.25] | 0.204 |
| Cr* (umol/L), median (Q1, Q3) | 73.00 [60.00;96.00] | 72.50 [61.00;97.00] | 76.00 [60.00;90.75] | 0.923 |
| eGFR* (ml/min/1.73m^2^), mean ± SD | 90.28 (33.51) | 89.65 (32.72) | 91.76 (35.34) | 0.532 |
| ALT* (U/L), median (Q1, Q3) | 21.55 [15.47;34.65] | 20.80 [15.40;33.30] | 21.90 [15.83;38.03] | 0.509 |
| AST* (U/L), median (Q1, Q3) | 48.85 [34.50;74.25] | 49.80 [34.42;74.05] | 47.80 [34.85;75.10] | 0.921 |
| TBIL* (umol/L), median (Q1, Q3) | 19.10 [12.70;29.02] | 19.20 [12.70;29.08] | 19.10 [14.05;28.45] | 0.832 |
| PT* (s), median (Q1, Q3) | 13.60 [12.88;14.53] | 13.70 [12.80;14.60] | 13.50 [12.90;14.50] | 0.565 |
| APTT* (s), median (Q1, Q3) | 32.55 [29.50;36.00] | 32.75 [29.80;36.08] | 32.15 [29.30;35.77] | 0.243 |
| TT* (s), median (Q1, Q3) | 17.30 [16.40;19.20] | 17.30 [16.40;19.48] | 17.25 [16.40;18.67] | 0.282 |
| FIB* (g/L), median (Q1, Q3) | 3.00 [2.50;3.50] | 3.00 [2.50;3.50] | 3.10 [2.60;3.60] | 0.319 |
| Drainage (ml)/100, median (Q1, Q3) | 16.70 [9.80;29.13] | 16.95 [9.96;29.28] | 16.30 [9.20;27.10] | 0.579 |
| Bridging number, median (Q1, Q3) | 1.00 [1.00;2.00] | 1.00 [1.00;2.00] | 1.00 [1.00;2.00] | 0.628 |
| Number of arterial grafts, n (%) |  |  |  | 0.633 |
| 0 | 149 (29.80%) | 103 (29.43%) | 46 (30.67%) |  |
| 1 | 320 (64.00%) | 222 (63.43%) | 98 (65.33%) |  |
| 2 | 29 (5.80%) | 23 (6.57%) | 6 (4.00%) |  |
| 3 | 2 (0.40%) | 2 (0.57%) | 0 (0.00%) |  |
| Number of vein grafts, n (%) |  |  |  | 0.615 |
| 0 | 201 (40.20%) | 147 (42.00%) | 54 (36.00%) |  |
| 1 | 251 (50.20%) | 170 (48.57%) | 81 (54.00%) |  |
| 2 | 47 (9.40%) | 32 (9.14%) | 15 (10.00%) |  |
| 3 | 1 (0.20%) | 1 (0.29%) | 0 (0.00%) |  |
| Duration of surgery (min), median (Q1, Q3) | 365.00 [315.00;425.00] | 370.00 [315.00;433.75] | 360.00 [306.25;418.75] | 0.298 |
| CPB time (min)/10, median (Q1, Q3) | 17.60 [14.45;21.83] | 17.50 [14.50;21.98] | 17.80 [14.22;21.60] | 0.815 |
| Ascending aortic block time (min), median (Q1, Q3) | 120.00 [90.00;150.00] | 120.00 [90.25;154.25] | 119.25 [89.25;148.75] | 0.961 |
| IB (ml)/10, median (Q1, Q3) | 100.00 [80.00;150.00] | 100.00 [80.00;150.00] | 100.00 [80.00;140.00] | 0.073 |
| IBT (ml), median (Q1, Q3) | 800.00 [0.00;1300.00] | 850.00 [226.25;1300.00] | 625.00 [0.00;1318.75] | 0.158 |

* = Postoperative laboratory indicators

BMI=body mass index, PCI=percutaneous coronary intervention, DVT=deep vein thrombosis, NYHA=New York Heart Association, LVEF=left ventricular ejection fraction, PLT=platelets, HB=hemoglobin, WBC=white blood cells, eGFR=estimated glomerular filtration rates, ALT=Alanine amio transferase, AST=Aspartate transaminase, TBIL=total bilirubin, BNP=B-type natriuretic peptide, PT=prothrombin time, APTT=activated partial thromboplastin time, TT=thrombin time, FIB=fibrinogen, Drainage/100= Postoperative drainage(ml)/100, CPB time/10= cardiopulmonary bypass time (min)/10, IB/10= Intraoperative bleeding (ml)/10, IBT= intraoperative blood transfusion

**Table S3. Bleeding risk factors identified through univariate logistic regression analysis with a p-value of less than 0.05.**

| Variables | B | OR（95%CI） | P |
| --- | --- | --- | --- |
| Gender | -0.837 | 0.433 (0.255-0.735) | 0.002 |
| Age | 0.041 | 1.041 (1.013-1.073) | 0.006 |
| Age>65 | 1.205 | 3.338 (1.846-6.371) | <0.001 |
| Diabetes | 1.344 | 3.833 (2.189-6.719) | <0.001 |
| Abnormal renal function | 1.829 | 6.227 (1.73-24.95) | 0.006 |
| Anemia | 1.776 | 5.905 (2.904-12.16) | <0.001 |
| AF | 0.676 | 1.966 (1.136-3.375) | 0.015 |
| HB | -0.031 | 0.97 (0.956-0.983) | <0.001 |
| Cr | 0.005 | 1.005 (1.001-1.009) | 0.021 |
| eGFR | -0.014 | 0.986 (0.977-0.995) | 0.003 |
| BNP | 0 | 1 (1-1.001) | 0.016 |
| PLT* | -0.008 | 0.992 (0.985-1) | 0.045 |
| HB* | -0.032 | 0.968 (0.95-0.986) | 0.001 |
| Cr* | 0.006 | 1.006 (1.001-1.011) | 0.019 |
| EGFR* | -0.016 | 0.985 (0.976-0.993) | <0.001 |
| Drainage /100 | 0.025 | 1.025 (1.014-1.037) | <0.001 |
| CPB time/10 | 0.078 | 1.081 (1.04-1.126) | <0.001 |
| IB/10 | 0.008 | 1.008 (1.004-1.012) | <0.001 |
| IBT | 0.001 | 1.001 (1-1.001) | <0.001 |

* = Postoperative laboratory indicators

AF=Atrial fibrillation, HB=hemoglobin, eGFR=estimated glomerular filtration rates, BNP=B-type natriuretic peptide, PLT=platelets, Drainage/100= Postoperative drainage(ml)/100, CPB time/10= cardiopulmonary bypass time (min)/10, IB/10= Intraoperative bleeding (ml)/10, IBT=intraoperative blood transfusion

**Table S4. The results of the multivariate logistic regression analysis, based on bleeding risk factors selected by LASSO regression.**

| Variables | B | OR（95%CI） | VIF | P |
| --- | --- | --- | --- | --- |
| Age>65 | 1.539 | 4.662 (2.202-10.69) | 1.187 | <0.001 |
| Diabetes | 1.697 | 5.457 (2.722-11.25) | 1.118 | <0.001 |
| Anemia | 1.571 | 4.813 (1.867-12.66) | 1.372 | 0.001 |
| AF | 0.879 | 2.409 (1.193-4.914) | 1.194 | 0.014 |
| Gender | -1.063 | 0.345 (0.170-0.680) | 1.340 | 0.002 |
| Cr | 0.004 | 1.004 (0.999-1.008) | 1.277 | 0.055 |
| Drainage/100 | 0.014 | 1.015 (1.001-1.027) | 1.155 | 0.02 |
| CPB time/10 | 0.078 | 1.081 (1.021-1.145) | 1.350 | 0.007 |
| IB/10 | 0.004 | 1.005 (1.000-1.009) | 1.333 | 0.045 |

AF= Atrial fibrillation, Drainage/100= Postoperative drainage(ml)/100, CPB time/10= cardiopulmonary bypass time (min)/10, IB/10= Intraoperative bleeding (ml)/10


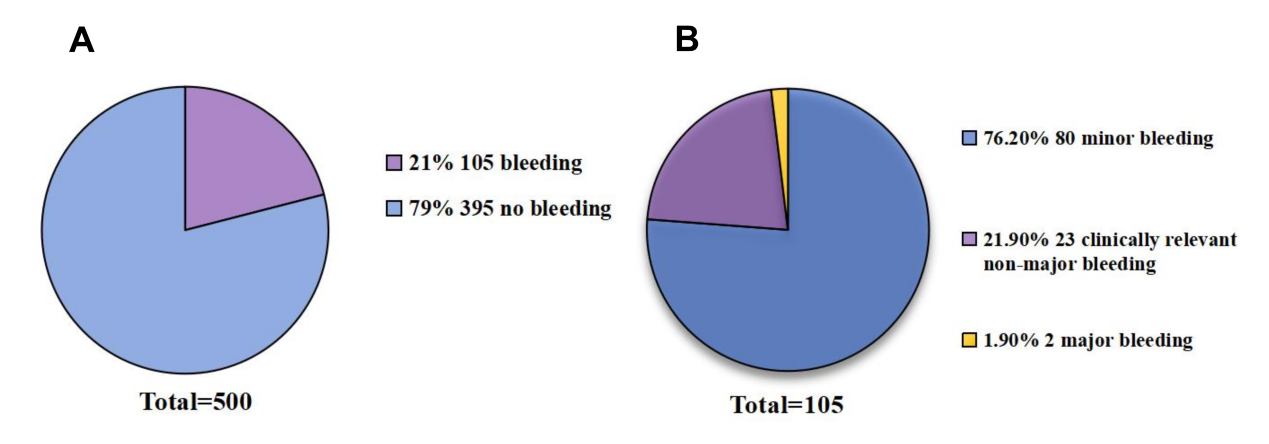


**Fig S1.** **Profile of bleeding events in patients receiving anticoagulation combined with antiplatelet therapy after cardiac surgery.**

1. Proportion of bleeding patients receiving anticoagulation combined with antiplatelet therapy after cardiac surgery.
2. Major types of bleeding after cardiac surgery receiving anticoagulation combined with antiplatelet therapy.

Case 1: A high-risk elderly patient with comorbidities

A 72-year-old male with a history of atrial fibrillation, diabetes, and hypertension underwent aortic valve replacement combined with coronary artery bypass grafting. Based on our nomogram, this patient was identified as having a high risk of bleeding due to multiple predictive factors, including advanced age, diabetes, atrial fibrillation, and a prolonged duration of extracorporeal circulation during surgery. The model indicated a high likelihood of postoperative bleeding, prompting the clinical team to adjust the postoperative anticoagulation and antiplatelet medication dosages, adopt more aggressive blood glucose control measures, and closely monitor the patient's hemoglobin levels. After discharge, the clinical pharmacist inquired about the patient's recovery and coagulation indicators such as the international normalized ratio weekly, guiding the patient to adjust the anticoagulation medication dosage in a timely manner. These proactive interventions, tailored specifically to reduce bleeding complications, ultimately prevented major bleeding and facilitated the patient's postoperative recovery.

Case 2: A low-risk young patient with minor comorbidities

A 45-year-old male with no significant medical history underwent mitral valve replacement combined with coronary artery bypass grafting. The nomogram identified this patient as having a low risk of postoperative bleeding based on factors such as age, no comorbidities, normal preoperative blood tests, intraoperative bleeding less than 800 ml, and low postoperative drainage volume. This low-risk classification allowed the clinical team to continue with standard antithrombotic therapy without additional interventions, thereby promoting smooth postoperative recovery and minimizing the need for monitoring bleeding complications.

These cases demonstrate how our nomogram serves as a powerful tool in clinical decision-making. By identifying individual patient risks, the model supports personalized treatment strategies, optimizes therapeutic regimens, and improves patient outcomes. The nomogram helps clinicians decide when to intensify monitoring or modify treatment plans to minimize complications, thereby enhancing patient care quality.
